# Identification of novel compound heterozygous mutations in the GLB1 gene by whole-exome sequencing in a case of infantile GM1 gangliosidosis: a case report

**DOI:** 10.3389/fped.2026.1839278

**Published:** 2026-05-22

**Authors:** Guoxing Zhong, Kaiping Wang, Kangrong Liao, Qiyuan Xie, Jianhong Chen, Rong Yu

**Affiliations:** 1 Department of Medical Genetics and Prenatal Diagnosis, Huizhou First Maternal and Child Health Care Hospital, Huizhou, Guangdong, China; 2 Department of Pediatrics Ward 3, Huizhou First Maternal and Child Health Care Hospital, Huizhou, Guangdong, China

**Keywords:** case report, *GLB1* gene, GM1 gangliosidosis, mutation, whole-exome sequencing

## Abstract

**Background:**

GM1 gangliosidosis was a rare, fatal autosomal recessive lysosomal storage disorder caused by biallelic mutations in the *GLB1* gene. Whole-exome sequencing (WES) was increasingly utilized to identify novel pathogenic variants in the *GLB1* gene among undiagnosed pediatric cases.

**Case presentation:**

We reported a 9-month-old male infant with developmental delay, hepatomegaly, extensive Mongolian spots, and hypotonia. WES identified two novel compound heterozygous GLB1 variants: a paternal c.792 + 1G > A splice-site mutation and a maternal c.1572_1573insC(p.Gly525Argfs*7) frameshift mutation. Both were classified as pathogenic by ACMG guidelines. *β*-galactosidase activity was markedly deficient, confirming the diagnosis. The family received genetic counseling and opted for prenatal diagnosis in a subsequent pregnancy. At age 2 years, the patient exhibited an inability to speak or walk and had a history of recurrent severe pneumonia requiring multiple hospitalizations, with his overall condition currently managed supportively.

**Conclusions:**

Two novel pathogenic GLB1 mutations expanded the mutational spectrum of infantile GM1 gangliosidosis. WES with enzymatic validation enabled precise diagnosis, genetic counseling, and prenatal management. The development of targeted therapies remained imperative to alter the disease’s natural course.

## Background

GM1 gangliosidosis was a rare autosomal recessive lysosomal storage disorder with an estimated incidence of 1 in 100,000 to 200,000 newborns (1). The clinical presentation of GM1 gangliosidosis, a progressive neurodegenerative disease involving visceral and skeletal systems due to substrate accumulation, was divided into three phenotypes (infantile, late-infantile/juvenile, and adult) according to the age of onset and the severity of neurological symptoms (2). This disease was caused by mutations in the human *β*-galactosidase (*GLB1*) gene, which led to a deficiency in the enzyme *β*-galactosidase (3). The *GLB1* gene, which was associated with metabolic disease and located on chromosome 3 at 3p21.33, encoded the lysosomal enzyme *β*-galactosidase and elastin-binding protein (EBP) (4). Mutations in *GLB1* could cause two different lysosomal storage diseases: GM1 gangliosidosis and Morquio B disease (5). The broad implementation of WES as a diagnostic tool for pediatric rare diseases facilitated the discovery of a growing number of novel pathogenic mutations in the *GLB1* gene (6). In the current study, we described the first case of infantile GM1 gangliosidosis caused by rare, novel compound heterozygous mutations in the *GLB1* gene, which were identified by WES and shown to be inherited from the parents.

## Case presentation

**Figure 1:**
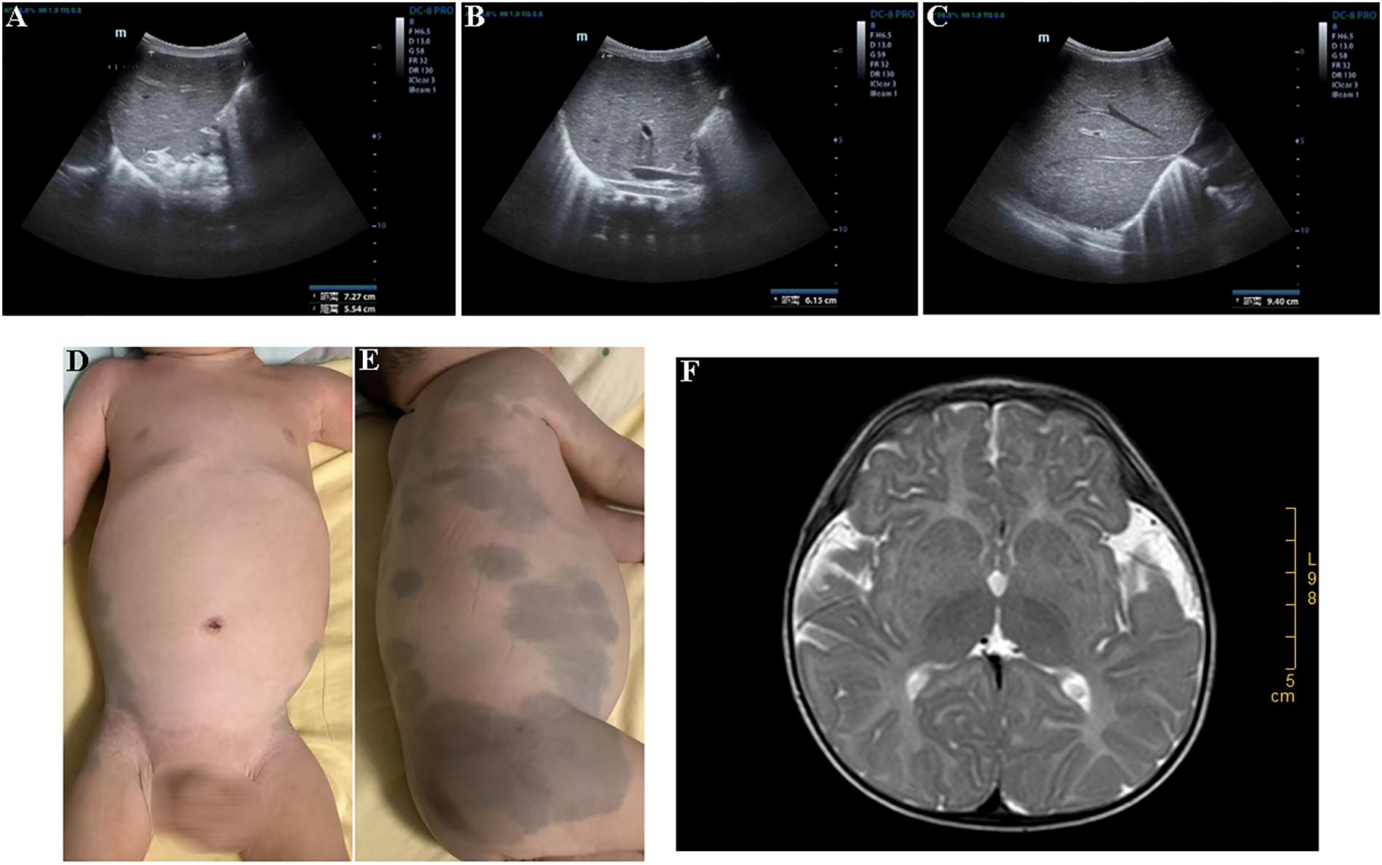
Clinical and imaging findings of the proband. **(A–C)** Abdominal ultrasonography at 11 months of age demonstrated hepatomegaly, indicated by measurements of the left liver lobe (longitudinal diameter 72.7 mm, anteroposterior diameter 55.4 mm), a subcostal extension of the liver edge of 61.5 mm, and a right liver oblique diameter of 94 mm. **(D,E)** Physical examination at 1.5 years of age revealed a protuberant abdomen and extensive slate-gray Mongolian spots located on the right lumbar-gluteal region and extending from the left lumbar-gluteal region to the upper thigh. **(F)** Brain MRI at 9 months of age revealed delayed myelination.

**Figure 2:**
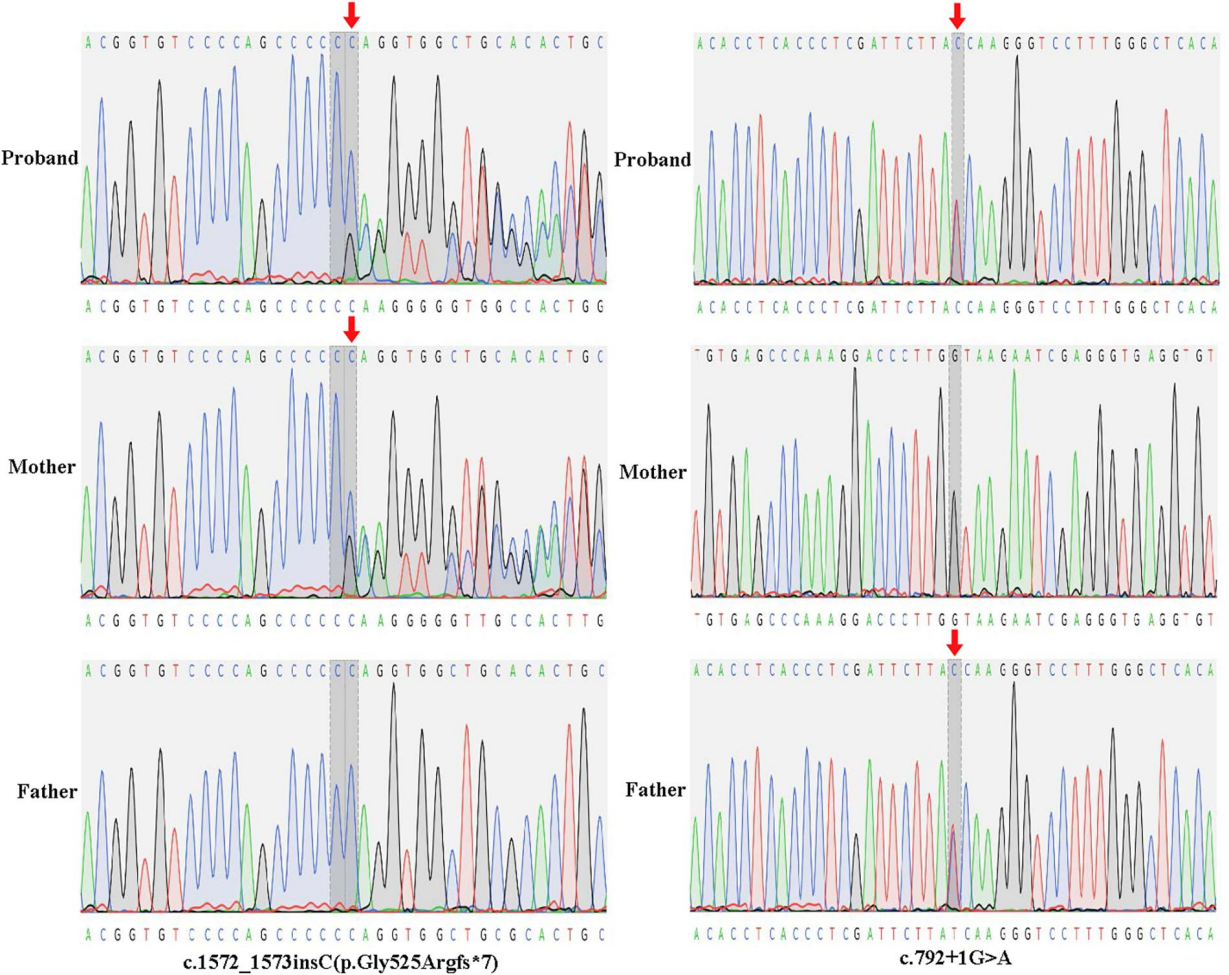
Sanger sequencing validation of the WES results. Sanger sequencing confirmed that the c.1572_1573insC (p.Gly525Argfs*7) variant was maternally inherited and the c.792 + 1G > A splice-site variant was paternally inherited.

The proband was a male infant born at 37 weeks of gestation via cesarean section, with a birth weight of 3.2 kg and a length of 50 cm. At birth, bilateral scrotal enlargement (each ∼3.5 × 2.5 cm) was noted. Ultrasound at 8 months confirmed bilateral hydrocele testis, for which surgical intervention was performed. The proband had not undergone routine developmental surveillance. At 9 months and 28 days of age, his Gesell Developmental Scale score was 29. His weight was 7.85 kg, length 70 cm, and head circumference 43 cm. He was unable to roll over, had poor head control, could not sit with support, and did not bang toys. Although he recognized familiar faces, he did not babble “baba” or “mama.” Subsequently, he was hospitalized due to developmental delay, hepatomegaly (Figures 1A–D), extensive slate-gray Mongolian spots (Figures 1D,E), hypotonia, and reduced muscle strength. A series of diagnostic tests were conducted, including an electroencephalogram (EEG), magnetic resonance imaging (MRI), and WES. EEG findings were unremarkable. However, brain MRI at 9 months of age showed indistinct gray-white matter boundaries in the bilateral frontoparietal lobes, decreased T1WI signal and increased T2WI/FLAIR signal in the bilateral basal ganglia and centrum semiovale, consistent with delayed myelination (Figure 1F). WES identified two novel compound heterozygous mutations in the *GLB1* gene, which were inherited from the parents: the c.1572_1573insC (p.Gly525Argfs*7) variant was of maternal origin, and the c.792 + 1G > A splicing variant was of paternal origin. To confirm the mutation sites, Sanger sequencing of the *GLB1* gene was performed (Figure 2). And neither variant had been previously reported in the literature. According to the ACMG guidelines, both variants were classified as pathogenic (Table 1). Following the genetic findings, a sphingolipidosis enzyme assay was performed on the proband. Enzymatic analysis of the proband revealed a definitive deficiency of *β*-galactosidase activity (1.1 nmol/mg.h; reference range 50–140), while the activities of other lysosomal enzymes, including arylsulfatase A, *β*-galactocerebrosidase, and *β*-hexosaminidases, were within normal limits (Table 2). This biochemical profile provided confirmatory evidence for the diagnosis of infantile GM1 gangliosidosis, consistent with the identified *GLB1* mutations. Based on the aforementioned genotypic and clinical phenotypic findings, the proband was ultimately diagnosed with infantile GM1 gangliosidosis caused by two rare and previously unreported compound heterozygous *GLB1* mutations inherited from the parents. When the proband was one and a half years old, his mother became pregnant again. Chorionic villus sampling was subsequently performed, followed by chromosomal microarray analysis and Sanger sequencing for validation. Unfortunately, the fetal genotype recapitulated the proband's compound heterozygous *GLB1* mutations. Additionally, a 444-kb pathogenic copy number variation on chromosome 15 (15q11.2) was unexpectedly identified. Following genetic counseling, the parents made the decision to terminate the pregnancy. Given that the parental pathogenic alleles in the *GLB1* gene were known now, preimplantation genetic testing for monogenic disorders (PGT-M) in conjunction with invasive prenatal diagnosis (IPD) was recommended for any future pregnancies to prevent the inheritance of both pathogenic variants. A retrospective review of the proband's prenatal records revealed that, at 32 weeks of gestation, fetal ultrasound findings included ascites, bilateral hydrocele, nuchal fold (NF) thickening, and an increased cardiothoracic area ratio. Considering the gestational age and other factors, the pregnant woman declined further invasive prenatal diagnostic procedures and opted to continue the pregnancy. At the current age of 2 years, the proband had been hospitalized at our institution six times since birth due to severe pneumonia, with each episode managed with appropriate symptomatic treatment. The patient currently presented with an inability to speak or walk. His overall condition was now stable, and he continued to be monitored in our follow-up program.

**Table 1:** ACMG pathogenicity assessment of this two novel *GLB1* variants.

No.	Variant Description	Variant Type & Predicted Consequence	ACMG Evidence Codes Applied	Pathogenicity Classification
1	c.1572_1573insC (p.Gly525Argfs*7)	Frameshift insertion → Premature stop codon (Null allele)	PVS1, PM2, PP4	Pathogenic
2	c.792 + 1G > A	Canonical splice-site variant → Aberrant splicing (Null allele)	PVS1, PM2, PP4	Pathogenic

*7 means: after the frameshift, the 7th codon in the new reading frame becomes a stop codon (*), causing the protein to be truncated prematurely.

**Table 2:** Enzymatic activity profile in the proband.

No.	Enzyme Assayed	Result	Unit	Reference Range
1	*β*-Galactosidase	1.1 ↓	nmol/mg.h	50–140
2	Arylsulfatase A	329.3	nmol/mg.17h	217.7–361.6
3	*β*-Galactocerebrosidase	37.9	nmol/mg.17h	18–75
4	*β*-Hexosaminidase (A&B)	2409	nmol/mg.h	600–3500
5	*β*-Hexosaminidase A	338.2	nmol/mg.h	150–365

## Discussion and conclusions

Previous studies had shown that the infantile form (Type I) of GM1 gangliosidosis was the most severe phenotype, presenting within the first year of life (often by 6 months) with rapid psychomotor regression, visceromegaly (particularly hepatosplenomegaly), macular cherry-red spots, coarse facial features, skeletal dysplasia, and growth retardation, typically leading to death before 3 years of age, often due to complications like recurrent bronchopneumonia (1–3, 7). Our case exhibited two distinctive features that deviate from the classic infantile GM1 gangliosidosis phenotype: the absence of macular cherry-red spots and the presence of extensive Mongolian spots. This presentation was consistent with a meta-analysis indicating that the cherry-red spot was not a universal finding, being observed in approximately 59% of patients (8). Furthermore, the prominent Mongolian spots in our proband aligned with the recognition that such extensive and persistent dermal melanocytosis could serve as an early diagnostic sign, often preceding other classical neurological features in the diagnostic workup of infant GM1 gangliosidosis (8, 9). The pathogenesis of extensive dermal melanocytosis in GM1 gangliosidosis is incompletely understood. Accumulated GM1 ganglioside may disrupt NGF-TrkA signaling, impairing melanoblast migration from the neural crest to the epidermis (10). Deficiency of EBP, an alternatively spliced product of *GLB1*, may further contribute to melanocyte persistence in the dermis (11). These mechanisms likely explain the atypical extent, persistence, and lack of spontaneous regression observed in these patients compared to common Mongolian spots. Currently, there were no FDA-approved disease-modifying therapies for GM1 gangliosidosis. Consequently, the management of affected patients was primarily limited to symptomatic and supportive care (8). This stark reality underscored the critical importance of preventive strategies, among which carrier screening and prenatal diagnosis were paramount (10). Classic prenatal findings of GM1 gangliosidosis include nonimmune hydrops fetalis (NIHF), intrauterine growth restriction, and placental vacuolization (10). Transient NIHF has also been documented (12). In the present case, fetal ultrasound at 32 weeks revealed ascites, bilateral hydrocele, NF thickening, and an increased cardiothoracic ratio, without overt hydrops. This distinct combination—particularly the bilateral hydrocele and NF thickening—had not been previously highlighted in GM1 gangliosidosis, likely reflecting the specific *GLB1* null genotype. These findings expanded the known prenatal spectrum and emphasized the need for genotype-phenotype correlation studies (1). Consequently, future research correlating specific *GLB1* genotypes with detailed prenatal imaging findings was crucial to refine prognostic accuracy and prenatal diagnostic criteria.

According to the publicly accessible ClinVar database (https://www.ncbi.nlm.nih.gov/clinvar/), more than 389 distinct mutations in the *GLB1* gene had been classified as pathogenic or likely pathogenic to date. In this study, genetic analysis revealed two novel heterozygous variants in the *GLB1* gene: a frameshift insertion, c.1572_1573insC, and a canonical splice-site mutation, c.792 + 1G > A. Both variants were absent from the Genome Aggregation Database (gnomAD v4.1). In silicoanalyses using SpliceAI, CADD, and MutationTaster also failed to retrieve predictions for these variants. According to the ACMG/AMP guidelines, both variants were classified as Pathogenic (see Table 1). The c.1572_1573insC variant was a clear null allele, expected to cause the creation of a premature stop codon and result in a complete loss of functional protein. Similarly, the c.792 + 1G > A variant was predicted to severely disrupt mRNA splicing, also leading to a loss-of-function allele. The compound heterozygous state of these two pathogenic variants, resulting in a almost all loss of *β*-galactosidase function, confirmed the molecular etiology and explained the severe infantile phenotype observed in our proband.

In conclusion, this study identified two novel compound heterozygous mutations (c.1572_1573insC and c.792 + 1G > A) in the *GLB1* gene as the definitive cause of severe infantile GM1 gangliosidosis, thereby expanding the known mutational spectrum of the disease. Our findings robustly confirmed the genotype-phenotype correlation wherein these null alleles precipitate a near-total loss of *β*-galactosidase activity, consistent with the classic Type I phenotype. The precise molecular diagnosis, achieved through WES and ACMG guidelines, was indispensable for providing definitive genetic counseling and enabling informed reproductive choices for the family. Looking forward, future studies correlating specific *GLB1* genotypes with their detailed prenatal imaging findings and long-term clinical progression are essential to refine diagnostic criteria and improve patient management. The hope for the future lies in the development of targeted therapies, such as the gene therapy candidates currently under investigation, to alter the devastating natural history of this severe disorder (13).

## Data Availability

The original contributions presented in the study are included in the article/supplementary material, further inquiries can be directed to the corresponding author/s.
